# Suicidal ideation and suicide attempts in subjects aged 15–19 in Lomé (Togo)

**DOI:** 10.1186/s13104-019-4233-0

**Published:** 2019-03-29

**Authors:** Tchin Darré, Koffi Assaba Cinthia Consuela, Bayaki Saka, Toukilnan Djiwa, Koumavi Didier Ekouévi, Gado Napo-Koura

**Affiliations:** 1 Department of Pathology, University of Lomé, B.P. 1515 Lome, Togo; 2 Department of Dermatology, University of Lomé, Lome, Togo; 3 Department of Health and Epidemiology, University of Lomé, Lome, Togo

**Keywords:** Suicides, Adolescents, Lomé (Togo)

## Abstract

**Objectives:**

The purpose of this study was to study the epidemiological and clinical profile of adolescents with suicidal thoughts, with or without suicide attempts, and to identify associated factors.

**Results:**

A total of 155 (16.5%) of the 941 adolescents interviewed had suicidal thoughts. The average age of the respondents was 18 ± 2.1 years. The sex ratio (m/f) was 1.4. With regard to marital status, 70.2% were single and 29.8% were in a relationship with a cohabiting partner. Family history of suicide was reported in 40%. In their personal history, eight were infected with HIV, three were chronic ethylic and two were diabetics. Forty-six (29.7%) of the 155 adolescents who had suicidal ideation had ever had a suicide attempt. Teens affected by suicide lived in a boarding school in 25.8%, with one parent in 23.9% and 50.3% with both parents. Factors associated with suicide attempts were female sex (p = 0.0107), age over 18 years (p = 0.0177), living in a couple (p = 0.0316), underlying immunodepression (HIV infection, p = 0.0059, sickle cell disease, p = 0.0043) and having a family history of suicides (p = 0.0461).

## Introduction

Worldwide, suicide is a common cause of death among young people, which is a major public health problem. It is the second leading cause of death after road accidents among young people aged 15–19 in Western countries [1, 2]. Adolescence is a period more likely to lead to suicidal behavior [3]. It is important to know that this is an intense period of social, family, physical and emotional change [4]. In sub-Saharan Africa in general, and in Togo in particular, suicide attempts remain a poorly evaluated subject due to socio-cultural considerations and lack of a longitudinal approach [4, 5]. The objective of this study was to determine the prevalence and to study the sociodemographic and clinical characteristics of adolescents with suicidal ideation and suicide attempts in the adolescent population of Lomé (Togo).

## Main text

### Methodology

It was a descriptive cross-sectional study conducted among adolescents aged 15–19 enrolled in high schools in the city of Lomé and at the University of Lomé. It took place from March to June 2018 for duration of 3 months. Included were adolescents aged between 15 and 19 years enrolled in high schools in the city of Lomé and students aged 15–19 enrolled during the academic year 2017–2018 at the University of Lomé. The variables studied were: socio-demographic variables, motives for ideas and suicide attempts, means of suicide attempts.

The data was recorded on the Epidata 3.1 software. The data analysis was done on the software R in version 3.4.3. For the descriptive analysis, the quantitative variables were presented as mean and standard deviation and the qualitative variables as numbers and percentages. For the comparative analysis and the univariate analysis the threshold of significance was 0.05. The statistical tests used for the comparative analysis were the Chi-2 or Fisher test for the comparison of two qualitative variables and the Student’s test for the comparison of two means.

### Results

During this period, 941 teenagers accepted the questionnaire, 602 high school students and 339 students. Of these, 155 (16.5%) had suicidal thoughts. The average age of the subjects was 17.5 years and the sex ratio (M/F) was 1.4. Subjects with suicidal thoughts were high school students and secondary school students in 51.6% and 48.4% respectively. With regard to marital status, 71% (n = 106) were single, 28% (n = 43) were married couples with one partner and 1% (n = 2). The epidemiological data of adolescents with idea and attempted suicide are summarized in Table 1. The revelation of the intention to commit suicide was noted in 61 subjects (39.4%), of which 56 subjects to close and 5 subjects to a doctor. Family history of suicide in at least one parent was observed in 25.8% (n = 40). One hundred and one parents (65.2%) for whom we have information concerning their socio-economic conditions was of categories: weak in 49 cases, average in 41 and high in 11 cases. Forty-six (31 girls and 15 boys) or 29.7% of the subjects had already attempted suicide. The suicide idea/suicide attempt report was 3.4. Of his 46 subjects, 16 were followed by medical staff, 13 by a psychologist and 3 by a psychiatrist. Thirteen adolescents (8.4%) had an immunosuppression factor: an HIV infection was known in 8 subjects; 3 subjects were chronic ethyl and 2 subjects were diabetic. Subjects affected by the idea of suicide lived in a boarding school in 25.8% (n = 40) of cases, with one parent in 23.9% (n = 37) of cases and with both parents in 50.3% (n = 78) of cases with both parents. Factors associated with suicide attempts were female sex (p = 0.0107), age over 18 years (p = 0.0177), living in a couple (p = 0.0316), underlying immunodeficiency (HIV infection, p = 0.0059, sickle cell disease, p = 0.0043) and having a family history of suicides (p = 0.0461), (Table 1). The reasons for suicidal ideation were dominated by sentimental difficulties in 41 subjects and academic problems in 35 subjects (Table 2). The means of suicide were revealed by 43 subjects and dominated by drug poisoning (65.1%) (Table 3).

**Table 1:** Epidemiological characteristics of adolescents

Characteristics	Univariate model				
	n/N	%	OR	CI 95%	p value
Sex					0.0107
Male	12/65	18.5	1		
Female	34/90	37.8	2.68	[1.28–5.90]	
Median age (years)					0.0177
< 18	15/72	20.8	1		
≥ 18	31/80	38.8	2.40	[1.18–5.06]	
Level of study					0.6108
University	5/20	25.00	1		
Secondary	41/134	30.60	1.32	[0.48–4.28]	
Marital status					0.0316
Single	26/106	24.5	1		
Married	19/45	42.2	2.25	[1.07–4.72]	
Underlying immunodepression					0.0059
No	33/131	25.2	1		
Yes	13/24	54.2	3.51	[1.44–8.74]	
Sickles cells disease					0.0043
No	38/144	26.4	1		
Yes	8/11	72.7	7.44	[2.04–35.31]	
Live with family					0.2327
Intership	14/38	36.84	1		
Two parents	24/77	31.17	0.78	[0.34–1.78]	0.5434
Only one of the two	8/40	20.00	0.43	[0.15–1.16]	0.1026
Family history of suicide					0.0461
No	22/93	23.66	1		
Yes	24/62	38.71	2.04	[1.01–4.13]

**Table 2:** Motives for ideas and suicide attempts of adolescents in Lomé

Motives	Number of cases (n)	%
Sentimental difficulties	41	26.5
School problems	35	22.6
Health problems	14	9.0
Financial problems	13	8.4
Family problems	9	5.8
Death of a loved one	7	4.5
Loneliness	4	2.6
Unwanted pregnancies	4	2.6
Distaste of life	3	1.9
Absence of parents	2	1.3
Abstained	23	14.8
Total	155	100.0

**Table 3:** Means of suicide attempts of adolescents in Lomé

Means	Number of cases (n)	%
Drug poisoning	28	65.1
Ingestion of caustics	6	14.0
Hanging	4	9.3
Section of veins	3	7.0
Jumping from the top of a building	2	4.6
Total	43	100.0

### Discussion

This study shows that the most common reasons for teen ideation and suicide attempt are sentimental and academic difficulties and the most common suicide method used by adolescents is drug intoxication. It identified five factors associated with adolescent suicide attempts in Lomé, including female sex, older age, living in a couple, the existence of underlying immunosuppression, and having family history of suicide.

Among adolescents with suicidal attempts, there was a female predominance with thirty-four cases (p = 0.0107) with an odds-ratio of 2.68. In adolescents, suicidal acts are more often than not a means of escaping unbearable tension that the end point of a real desire for death [6]. In the United States, among the 15–19 year olds, 26% of boys and 41% of girls thought of suicide in 1997, in Quebec 21% of boys and 37% of girls [7]. Girls are more likely to attempt suicide; the teenage hospitalization rate for attempted suicide is double that of adolescents, but the suicide rate for boys aged 15–19 is four times higher than for girls in the same group age [5, 7]. The observed female predominance of suicides and suicide attempts can be explained by the early pubertal age in girls compared to boys, and the fact that boys have the opportunity to express their dissatisfaction or incomprehension by other means such as than delinquency or aggression, unlike women who have no alternative but to use violence [1, 3].

Thirty-one of the forty-six adolescents with suicidal attempts were between the ages of 18 and 19 (p = 0.0177), with an odds-ratio of 2.40. The advanced age of adolescents is a risk factor for suicidal attempts [2]. According to Boeninger et al. [8], the peak of suicidal behavior is around 16 years for girls and then decreases, while it continues to increase in boys up to 19 years of age. Twenty of the forty-six adolescents were married (p = 0.0316), with an odds-ratio of 2.25. Couple life would promote the occurrence of suicide attempts. In fact, sentimental difficulties dominate the motivation factors of suicidal ideas and attempts in our study as well as in the Ghanaian study where these difficulties are essentially the infidelity of the partner [9]. Thirteen of the adolescents who attempted a suicidal act had underlying immunodepression, particularly HIV infection, with an odds-ratio of 3.51. Adolescents with underlying immunosuppression would be about four times more likely to attempt suicidal acts compared to healthy adolescents [10]. This finding is explained by a lack of psychosocial care for people living with HIV [10, 11]. Twenty-four adolescents with suicidal attempts, had a family history of suicides (p = 0.0461), an odds-ratio of 2.04. Adolescents with a family member who has attempted suicide are more likely to be exposed [12].

## Limitations

The short duration of the study is an insufficiency of our work mainly related to a lack of funding. This lack of funding still explains the fact that the study was conducted in the city of Lomé alone and not on the whole territory that would have allowed to have a larger sample. In addition, it was difficult to verify the sincerity of the information provided by the adolescents surveyed.

## Abbreviation

HIV: human immonodeficiency virus
